# Effect of knee unloading shoes on regional plantar forces in people with symptomatic knee osteoarthritis – an exploratory study

**DOI:** 10.1186/s13047-018-0278-x

**Published:** 2018-06-26

**Authors:** Joyce A. C. van Tunen, Kade L. Paterson, Tim V. Wrigley, Ben R. Metcalf, Jonas B. Thorlund, Rana S. Hinman

**Affiliations:** 1 Research Unit for Musculoskeletal Function and Physiotherapy, Department of Sports Science and Clinical Biomechanics, University of Southern Denmark, Campusvej 55, 5230 Odense, Denmark; 2 Centre for Health, Exercise and Sports Medicine, Department of Physiotherapy, School of Health Sciences, The University of Melbourne, Parkville, VIC Australia

**Keywords:** Knee osteoarthritis, Foot, Unloading, Footwear, Pressure, Force

## Abstract

**Background:**

Knee ‘unloading’ footwear can reduce the external knee adduction moment in people with knee osteoarthritis, yet effects of these shoes on regional plantar forces are unknown. We evaluated the effects of unloading shoes on in-shoe regional plantar forces, and whether measures of foot posture and/or mobility moderate these effects in people with symptomatic knee osteoarthritis.

**Methods:**

In this exploratory study 21 participants underwent testing while wearing knee unloading shoes (ASICS GEL-Melbourne OA) and conventional shoes in random order. Peak total forces were compared across conditions for: lateral heel, medial heel, lateral forefoot, and medial forefoot. Arch index, centre of pressure position and medial-lateral heel peak force ratio were also evaluated. Foot posture, foot mobility magnitude and navicular drop were separately added to the mixed linear model to investigate if these modified the effect of footwear on outcomes.

**Results:**

Unloading shoes significantly increased lateral heel and lateral forefoot force (12.9 and 20.2% respectively, all *P* < 0.001), with concurrent decreases in the medial heel (8.9%, *P* = 0.001) and medial forefoot (9.9%, *P* = 0.005). Unloading shoes significantly shifted the centre of pressure anteriorly (4.7%, *P* < 0.001) and laterally (5.6%, *P* = 0.034), but did not affect the arch index (8.7%, *P* = 0.093). Foot posture, foot mobility magnitude and navicular drop did not moderate the effect of footwear on outcomes.

**Conclusion:**

Compared to conventional shoes, unloading shoes caused a lateral shift in foot pressure and force patterns. Although these effects were not moderated by foot posture, FMM or navicular drop, variability in the individual increases in lateral heel force suggests participant characteristics other than foot posture may play a role.

**Trial registration:**

ACTRN12613000851763. Registered 02 August 2013.

## Background

Knee osteoarthritis (OA) is a leading cause of pain and disability among older adults, with more than 200 million people affected worldwide [1]. As no cure exists for OA, current treatment is restricted to managing symptoms and improving function in an effort to maximise quality of life. In particular, self-management is fundamental, and clinical guidelines advocate appropriate footwear as important [2, 3].

Knee joint loading during walking is higher in the medial compared to the lateral compartment, which likely explains why knee OA occurs most commonly in the medial tibiofemoral joint. Increased medial knee joint loading, most commonly inferred using the external knee adduction moment (KAM), has been associated with greater pain and disability in people with knee OA [4], and worsening structural changes over time [5]. Accordingly, biomechanical strategies designed to reduce knee loading, such as knee braces, lateral wedges and unloading footwear, have received support in international guidelines for the management of people with knee OA [2, 3].

Unloading footwear with midsoles that are stiffer laterally than medially have been shown to reduce the KAM in those with knee OA, primarily by reducing the knee ground reaction force (GRF) lever arm (mainly via a lateral shift in the centre of pressure (CoP) under the foot) and the magnitude of the frontal plane GRF [6]. These findings suggest that reductions in the KAM with unloading shoes are associated with changes evident at the foot. However, research has only measured loads at the shoe-ground interface using force plates; no study to date has evaluated the effect of unloading shoes on in-shoe regional plantar pressures. Research on laterally wedged insoles (that are inserted into the patient’s own footwear) suggests they increase lateral pressures [7–10] and it is reasonable to hypothesise that unloading shoes may have a similar effect.

The actual effect of unloading shoes on changes in the KAM is quite variable across individuals [11, 12]. Identification of subgroups of “biomechanical responders” is an important OA research priority [3]. As such, measurement of regional plantar forces permits an increased understanding of how unloading footwear changes regional plantar loading, and may yield important insights into individual responses to this treatment. People with knee OA have a more pronated static foot posture [13] compared to those without knee OA, and concurrent foot pain is common in people with knee OA [14]. A pronated foot [15] and greater rear foot mobility [16] have been shown to be associated with greater reductions in the KAM with lateral wedge insoles. There is also some evidence that foot posture is associated with plantar pressures during walking [17]. Thus, it is possible that people with a more pronated foot posture, or greater foot mobility, experience different changes in plantar forces with unloading shoes. This information may help to expand our understanding of how unloading shoes influence foot and ankle biomechanics, and may also help explain why some people experience foot/ankle pain when wearing these types of shoes.

The aims of this exploratory study were to evaluate i) the effects of unloading shoes on in-shoe regional plantar forces, relative to conventional shoes without unloading features and; ii) whether measures of foot posture and/or foot mobility moderate these effects, in people with symptomatic medial knee OA.

## Methods

### Participants

Participants in this study were a sample of convenience that comprised a subset of volunteers enrolled in a 6-month randomised controlled trial comparing the effects of unloading and conventional walking shoes on knee OA symptoms [18]. The number of participants in this study was dictated by the number of participants in the control group (conventional shoes) whom were willing to undergo in-shoe plantar pressure data collection upon exit from the trial. For this exploratory cross-sectional study, 21 participants were recruited after completing the final 6-month assessment for the randomised controlled trial. Participants were included in the trial if they were aged 50 years or older, had knee pain on most days of the previous month, reported average pain in the previous week of at least 4 out of 10 on an 11-point numerical rating scale (NRS) (with terminal descriptors of “no pain” and “worst pain possible”), had radiographic evidence of medial compartment knee OA (Kellgren-Lawrence (KL) grade ≥ 2), and had definite medial tibiofemoral osteoarthritis on radiography. Exclusion criteria for the trial have previously been published [18]. Study procedures were approved from the University of Melbourne Human Research Ethics Committee (HREC No. 1239045) and all participants provided written informed consent.

### Unloading and conventional (control) shoes

**Fig. 1:**
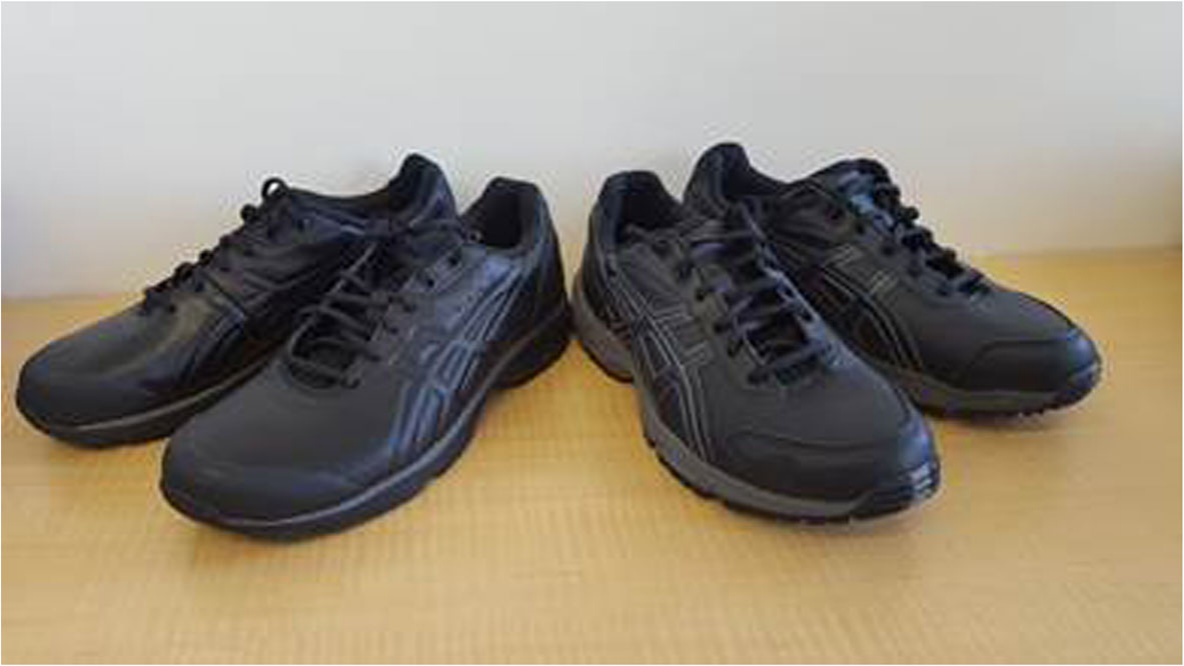
Conventional walking shoes (GEL-Odyssey; left) and unloading shoes (GEL-Melbourne OA; right) used in the study. Both shoes were visually similar

The unloading shoes were black leather commercially available unloading walking shoes (GEL-Melbourne OA [ASICS Oceania]) with triple-density midsoles that were stiffer laterally (Shore A rating of 65) than centrally (Shore A rating 55) and medially (Shore A rating of 45), and with mild (5-degree) lateral-wedge insoles attached to the underside of the sockliners (Fig. 1) [11]. The GEL-Melbourne OA weighed 334 g (based on women’s shoes size 8US). The conventional shoes were black leather commercially available neutral walking shoes (GEL-Odyssey [ASICS Oceania]) that were visually similar to the unloading shoes but possessed a mono-density midsole (Shore A rating of 55) and did not contain the lateral wedge. The GEL-Odyssey weighed 289 g (based on women’s shoes size 8US). During testing for this study, participants were not informed which shoes were the unloading shoes.

### Force measurements

An in-shoe plantar pressure measurement system (pedar-X insoles, version 24.3.5 software, Novel gmbh, Munich, Germany) was used to record regional plantar pressures for both feet at 100 Hz [19]. Each insole had 99 capacitive cells distributed throughout the insole.

Participants completed walking trials at their own self-selected speed in the unloading and conventional shoes, presented in random order, over an 8-m walkway. Two sets of photoelectric timing gates four meters apart were used to ensure walking speed was maintained within 5% of each participant’s average. For each shoe condition, data collection commenced approximately two meters after the beginning and before the end of the walkway to ensure steady state gait. Three practice trials were permitted for each shoe condition and discrete data from all recorded stance phases of the affected limb (usually 2–3) of the fourth trial were averaged per participant. For participants with bilateral knee symptoms, data from the most symptomatic knee was collected.

Insole pressure data from every individual cell were exported into custom Matlab (Mathworks Inc., USA) code, and converted from pressure (1 kPa = 0.1 N/cm^2^) to force for analysis, using the known (different) area of each cell: force (N) = pressure (N/cm^2^) x cell area (cm^2^). Medial and lateral sub regions were analysed. For these, the foot was initially separated into three regions: forefoot (40% of the total foot length), midfoot (30% of foot length) and heel (30% of foot length) [20]. The toe sub region was disregarded, because data from this region is highly variable [19]. To determine the medial and lateral regions of the heel and forefoot, we then divided each row of sensors in those regions in half. If a row contained an odd number of sensors, data from the centre sensors were disregarded.

**Fig. 2:**
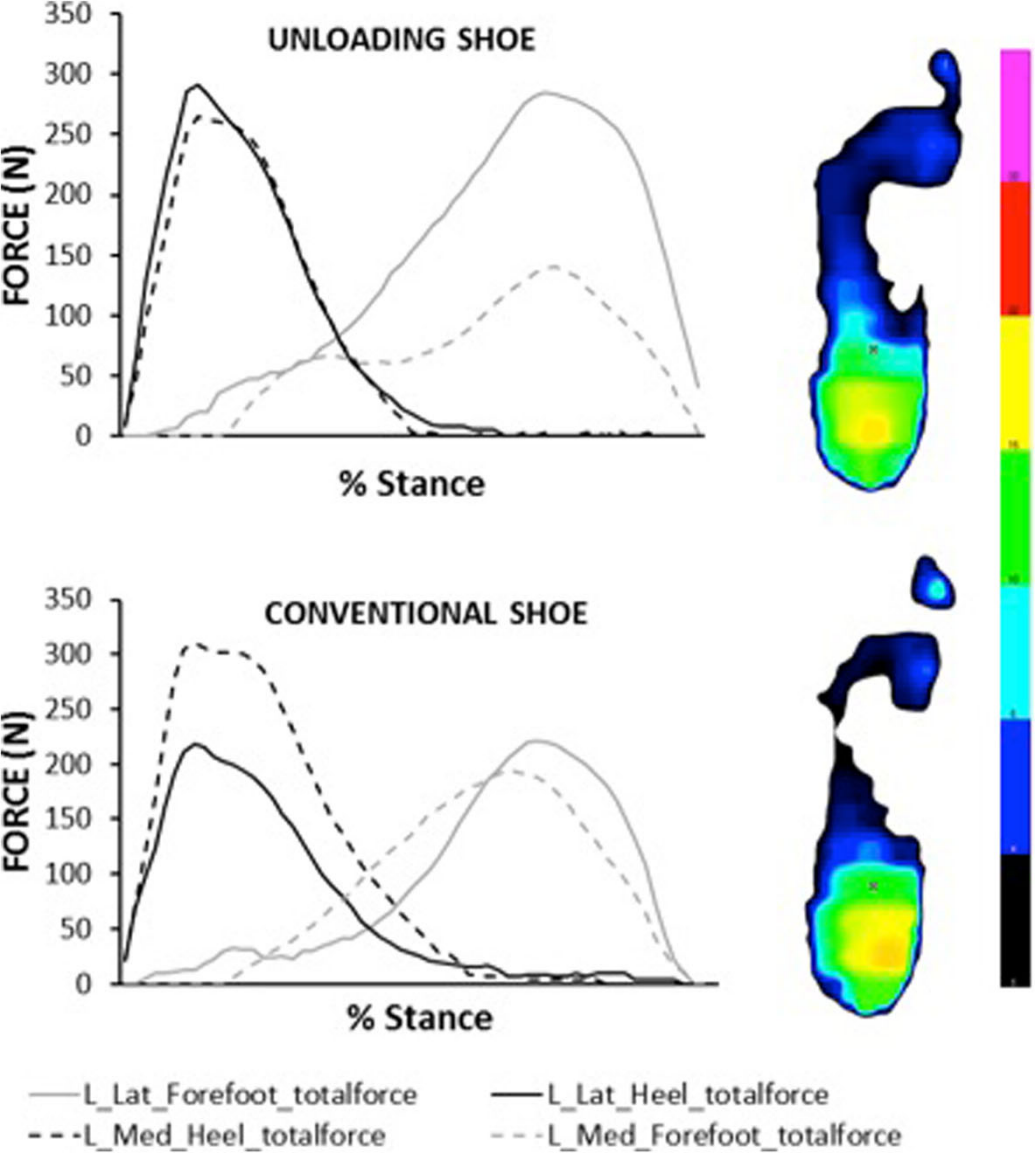
Exemplar subregional forces (N) for medial (dashed line) and lateral (solid line) subregions of the heel (black line) and forefoot (grey line), and their 2D pressure (N/cm^2^) maps at 25% stance. Colour map indicates pressure (N/cm^2^) from 1 (black) to 30 (pink)

Force across all cells in each sub region were summed at each point in time (Fig. 2). As force data was analysed paired across shoe conditions for each participant, no normalization for body size was applied. Centre of pressure data for the whole foot were exported separately via the FGT file format, as the manufacturer would not supply geometric locations of each sensor for CoP to be calculated. The insole XY coordinate system has its origin medial to the most posterior cell, with positive X direct laterally (through the base of the posterior cell), and positive Y anteriorly (through the most medial cell).

Peak total force (N) during stance was extracted for the following regions: lateral heel, medial heel, lateral forefoot, and medial forefoot. The ratio of the peak medial heel total force to the peak lateral heel total force during stance was also calculated [12]. Centre of pressure antero-posterior (x) and medio-lateral (y) positions (in mm) were extracted at 25% of stance phase [7]; this point was used because it roughly coincides with the first peak in the KAM [21]. The arch index was calculated as the ratio of midfoot loaded area relative to total loaded area excluding the toes [22], at midstance.

### Other descriptive measures

Age, sex, height and body mass were recorded, and body mass index (BMI, in kg/m^2^) was calculated. Participants reported symptom duration and laterality of symptoms. All participants underwent a semi-flexed weight-bearing postero-anterior knee radiograph to determine OA severity using the KL grading system (Grade 2 represents mild OA, Grade 3 moderate, and Grade 4 severe). Foot posture was assessed using the Foot Posture Index (FPI), a valid and reliable tool that scores six features of foot posture from − 2 (more supinated) to + 2 (more pronated) [23]. Total scores range from − 12 (highly supinated) to + 12 (highly pronated). Scores were categorised into highly supinated (< − 4), supinated (from − 4 to − 1), neutral (from 0 to + 5), pronated (from + 6 to + 9) and highly pronated foot posture (> + 9) [23]. Vertical and medial-lateral mobility of the midfoot was assessed with the foot mobility magnitude (FMM) [24], calculated as the square root of the sum of the squared difference in arch height from non-weight bearing to weight bearing and the squared difference in midfoot width from non-weight bearing to weight bearing. Foot mobility was also assessed using the navicular drop test, which is the difference in navicular height (in mm) between non-weight bearing and weight bearing [25]. Overall average pain while walking over the previous week was measured via an 11-point NRS [26]. Difficulty with physical functioning over the previous 48 h was measured by the function subscale of the Western Ontario and McMaster Universities Osteoarthritis Index (WOMAC, Likert version 3.1), with total scores ranging from 0 (no dysfunction) to 68 (maximum dysfunction) [27].

### Statistical analysis

Descriptive statistics are presented as means with standard deviations and numbers with proportions as appropriate. Mean differences in variables between shoe conditions were calculated, along with 95% confidence intervals. The main effect of ‘condition’ (i.e. unloading or conventional shoes) on variables was evaluated using a mixed linear random effects model with ‘condition’ as a fixed effect and ‘participant’ as random effect. To investigate if ‘foot posture’ modified the effect of shoes on plantar forces, we firstly dichotomised the FPI score as either neutral or pronated (pronated/highly pronated; no participant had a supinated or highly supinated foot posture). We then added foot posture to the mixed linear model as an additional fixed effect to test interaction between ‘condition’ and ‘foot posture’. Similarly, we also tested interaction between ‘FMM’ and ‘navicular drop’, respectively with shoe condition in separate models. FMM and navicular drop were added as continuous variables. All analyses were performed using STATA version 14.1, with an alpha level of less than 0.05 considered as statistically significant.

## Results

### Participant characteristics

Participant characteristics are presented in Table 1. Approximately half of the participants were female (57%). Participants were generally overweight [28] and mean symptom duration was 8 years. A range of radiographic disease severities was observed, with 29% of participants having mild, 43% having moderate, and 29% severe radiographic OA severity. Approximately half of the participants had a neutral foot posture (43%), and half had a pronated foot posture (57%) based on FPI score. Mean (SD) walking speed was not significantly different between conventional shoes (1.36 m/s (0.19)) and unloading shoes (1.35 m/s (0.20)).

**Table 1:** Characteristics of participants (*n* = 21)

Age, years	63.4 (7.0)
Male, n (%)	9 (43)
Symptom duration, years	8.0 (8.2)
Body mass index, kg/m^2^	29.8 (3.6)
Unilateral symptoms, n (%)	6 (29)
Radiographic disease severity grade, n (%)^a^	
Mild (Grade 2)	6 (29)
Moderate (Grade 3)	9 (43)
Severe (Grade 4)	6 (29)
Foot Posture Index classification, n (%)	
Normal (Scores from 0 to + 5)	9 (43)
Pronated (Scores from + 6 to + 9)	11 (52)
Highly pronated (Scores greater + 9)	1 (5)
Foot mobility magnitude, mm	9.6 (3.8)
Navicular drop, mm	7.6 (3.1)
Pain while walking (NRS)^b^	3.7 (3.0)
Physical function (WOMAC)^c^	18.5 (13.2)

Values are reported as mean (SD) unless otherwise indicated

^a^Using the Kellgren-Lawrence grading system; NRS = numerical rating scale; WOMAC = Western Ontario and McMaster Universities Osteoarthritis Index

^b^Ranges from 0 to 10 (higher scores indicate worse pain)

^c^Ranges from 0 to 68 (higher scores indicate worse function)

### Effects of unloading shoes on in-shoe regional plantar forces

**Fig. 3:**
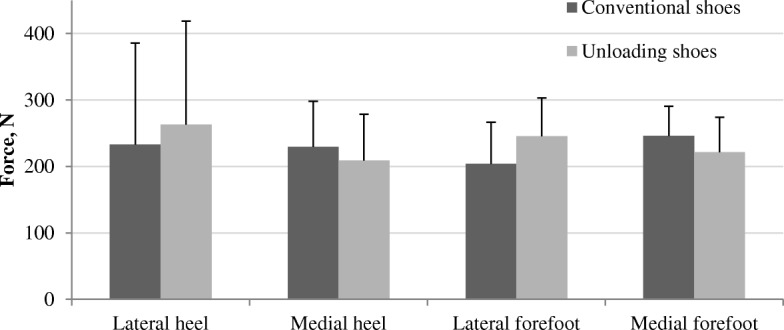
The effects of unloading shoes on regional foot forces. Data presented as mean (SD). All differences are statistically significant (*P* ≤ 0.005)

**Fig. 4:**
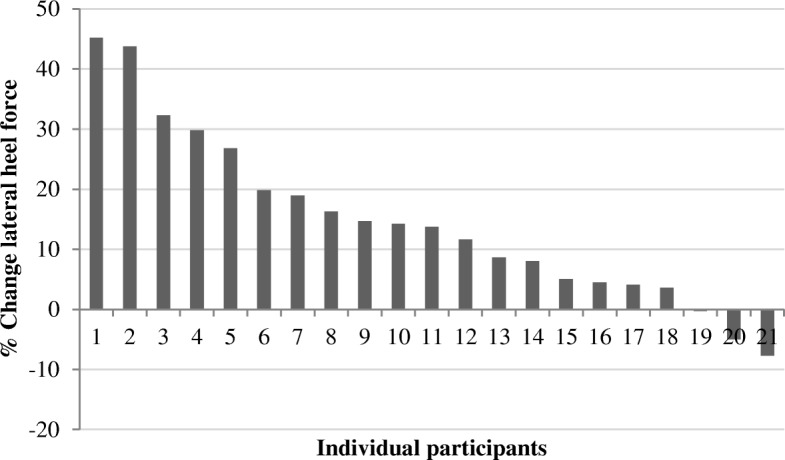
Individual changes in lateral heel force when walking in unloading shoes, reported as the percentage of change from the conventional shoes

The effects of unloading shoes on in-shoe regional plantar forces, relative to conventional shoes, are summarized in Table 2. Force in the lateral heel and forefoot regions significantly increased with unloading shoes (both *P* < 0.001), with corresponding decreases in the medial heel (*P* = 0.001) and forefoot (*P* = 0.005) force (Fig. 3). As a result of these changes, the medial-lateral heel force ratio also decreased significantly with unloading shoes (*P* < 0.001). Unloading shoes significantly shifted whole foot CoP anteriorly (*P* = 0.034) and laterally (P < 0.001). There was no difference in arch index between the shoes (*P* = 0.093). Whilst all but three participants demonstrated an increase in lateral heel force with unloading shoes relative to conventional shoes (Fig. 4), there was a large individual variation in response. However, FPI, FMM and navicular drop were not found to significantly moderate the effect of unloading shoes for any variable (Table 2).

**Table 2:** The effect of unloading shoes on plantar forces relative to conventional shoes, and the interaction between shoe condition and foot posture and/or mobility

Plantar force measurements	Conventional shoes, Mean (SD)	Unloading shoes, Mean (SD)	Difference (95%CI)	*P* value	Interaction (condition^*^foot posture), *P* value	Interaction (condition^*^ FMM), *P* value	Interaction (condition^*^ navicular drop), *P* value
Peak lateral heel force, N	233.0 (68.3)	263.0 (69.6)	30.0 (18.1 to 42.0)	**< 0.001**	0.457	0.308	0.199
Peak medial heel force, N	229.4 (62.5)	208.9 (57.7)	−20.5 (− 32.3 to − 8.7)	**0.001**	0.393	0.249	0.169
Peak lateral forefoot force, N	204.1 (44.4)	245.4 (52.3)	41.3 (21.5 to 61.0)	**< 0.001**	0.374	0.334	0.815
Peak medial forefoot force, N	246.1 (74.8)	221.8 (63.9)	−24.3 (−41.2 to −7.4)	**0.005**	0.155	0.453	0.071
Arch index (midstance)	0.23 (0.04)	0.21 (0.03)	−0.01 (−0.03 to 0.00)	0.093	0.744	0.118	0.318
CoP *x*-position (25% stance), mm	53.0 (5.0)	55.5 (4.9)	2.5 (1.4 to 3.6)	**< 0.001**	0.289	0.292	0.435
CoP *y*-position (25% stance), mm	78.9 (15.5)	83.3 (14.7)	4.4 (0.3 to 8.5)	**0.034**	0.718	0.875	0.786
Medial – lateral heel peak force ratio	1.0 (0.3)	0.8 (0.3)	−0.2 (−0.3 to −0.1)	**< 0.001**	0.175	0.117	0.051

*CoP* centre of pressure

*FMM* foot mobility magnitude

Bold values indicate statistical significance (*P* < 0.05)

## Discussion

This study evaluated the effects of unloading shoes on regional plantar forces in people with symptomatic medial knee OA, and whether alterations in these were moderated by measures of foot posture and/or mobility. The results of this exploratory study showed that unloading shoes increased lateral heel and forefoot force, with concurrent reductions in medial sub regional forces. In addition, unloading shoes shifted the CoP anteriorly and laterally, but did not significantly change the arch index. Individual increases in lateral heel force with unloading shoes were variable in magnitude, suggesting individual participant characteristics may influence biomechanical responses to unloading footwear. However, our results showed that foot posture, FMM and navicular drop did not significantly moderate the effect of unloading shoes on any outcome.

Although no previous study has examined changes in regional plantar forces when wearing unloading shoes in people with knee OA, other research has evaluated the effects of similar foot-based biomechanical interventions. Our findings are generally consistent with the effects of lateral wedge insoles that are inserted into the patient’s own footwear. Most research on inserted lateral wedges suggests they increase lateral plantar pressures. For example, Leitch et al. [7] assessed the effect of 4 and 8 degree lateral heel wedges in participants with and without knee OA, and found that lateral heel wedges increased lateral heel pressure, as well as lateral and anterior CoP displacement. An increase in lateral pressure and/or a lateral shift of the CoP with lateral wedge insoles has also been observed in other studies involving people with OA and healthy individuals [8–10]. However, Erhart et al. [12] found increased medial-to-lateral pressure ratios in healthy young adults walking in shoes with 4 and 8 degree laterally wedged shoe midsoles (i.e. not inserted wedges), in comparison to unwedged shoes.

Additionally, there is some research evaluating the effects of unloading shoes on CoP measured using force platforms. Kean et al. [6] observed a lateral shift in CoP in people with knee OA and in overweight asymptomatic individuals, which is consistent with our current findings using a foot-based measurement system to evaluate in-shoe plantar forces. In contrast, Jenkyn et al. [29] observed a medial shift of the CoP, measured using a force platform, in people with medial knee OA when walking in unloading shoes with a dual, more laterally dense midsole. Reasons for this difference in findings are uncertain. In summary, most findings suggest that current foot-based biomechanical ‘unloading’ interventions for knee OA redistribute plantar forces from medial to lateral, which likely contributes to a laterally shifted CoP; however even when other patterns are observed, group reductions in knee loading are generally found with all these interventions [6, 11, 29–33].

Despite some individual variability in how unloading shoes influenced regional plantar forces, we did not find that measures of foot posture or mobility moderated the effects of unloading shoes on regional plantar forces in our study. There may be several reasons for this. To increase the external validity of our findings and ensure relevance to clinical practice, we used simple static foot measures. However static foot posture is not a good indicator of dynamic foot motion [34], and although foot mobility and navicular drop are more dynamic measures, other variables such as peak or total rearfoot eversion, or rearfoot moments [15], during walking may have yielded different results. Our study involved a relatively small sample size, which yielded a limited spread of foot postures, and may mean we were underpowered to detect an interaction between footwear condition and foot posture, foot mobility or navicular drop on plantar forces. We found borderline significant interaction effects for navicular drop on the effect of unloading shoes for peak medial forefoot force and the medial-lateral heel peak force ratio. We interpret this as being a chance finding, because of the number of statistical tests used, and the lack of a consistent pattern across all outcome variables. Alternatively, it is possible that those measures genuinely do not moderate the effects of unloading shoes on regional plantar forces, and other individual characteristics may explain the variability in biomechanical effects of unloading shoes.

Our findings may have clinical implications. Adverse effects at the foot/ankle with unloading shoes have been reported by some people with knee OA. In our recent clinical trial, 20% of participants reported foot/ankle pain, with 4% discontinuing treatment because of these adverse effects [18]. Importantly, the trial excluded people who reported ankle/foot pain in the previous 6 months, thus it is possible that this study may have underestimated the potential for foot-related adverse effects if people with existing foot and/or ankle problems were to wear such shoes. We observed small increases in lateral forefoot and lateral heel forces with unloading shoes, which may warrant caution with their use in people with concurrent foot/ankle pathology and/or symptoms. As concurrent foot pain is common in people with knee OA [14], unloading shoes could exacerbate foot problems in this subset of patients.

There are some limitations to this study. First, we only assessed the immediate effect of unloading shoes on plantar forces. Second, although we averaged data over multiple footstrikes, only data from a single walking trial was analysed. Third, our relatively small sample size means that our findings are only generalizable to people with knee OA who have characteristics similar to our sample. Further, although foot posture and mobility did not moderate effects of the shoes, other participant characteristics (such as KL-grade and knee alignment) may play a role. Last, this study only examined the effect of unloading shoes on regional plantar forces, in isolation from other lower limb biomechanical parameters. Future studies should combine regional plantar pressure measurements with gait analysis to determine whether changes in plantar forces are associated with altered knee biomechanics relevant to knee OA, such as the KAM.

## Conclusions

In conclusion, this exploratory study evaluated the immediate effects of unloading shoes on plantar force measurements, and whether measures of foot posture and/or mobility moderated these effects, in patients with medial knee OA. Findings demonstrated greater lateral forces with unloading shoes in rear- and forefoot sub regions, with a concurrent decrease in medial forces. Although effects of unloading shoes on plantar forces were not moderated by foot posture, FMM or navicular drop, variability in the individual increases in lateral heel force suggest other individual participant characteristics apart from foot posture may play a role.

## Abbreviations

CoP: Centre of pressure
FMM: Foot mobility magnitude
FPI: Foot posture index
GRF: Ground reaction force
KAM: Knee adduction moment
KL: Kellgren-Lawrence
NRS: Numeric rating scale
OA: Osteoarthritis
RCT: Randomized controlled trial
WOMAC: Western Ontario and McMaster Universities Osteoarthritis Index
